# A unique case of rectal cancer with coexistence of multiple pathways of carcinogenesis

**DOI:** 10.1186/s12957-023-03157-9

**Published:** 2023-09-01

**Authors:** Nisha Rateria, Ritu Ojha, Mridula Shukla, Manoj Pandey

**Affiliations:** 1 Department of Surgical Oncology, Institute of Medical Sciences, Banaras Hindu University, Varanasi, 221005 India; 2 Department of Radiodiagnosis, Institute of Medical Sciences, Banaras Hindu University, Varanasi, 221005 India; 3 Regional Research Lab, Lal Pathology, Shivpur, Varanasi India

## Abstract

**Background:**

Colorectal cancer with a global incidence of 10% has multiple pathways implicated in its carcinogenesis. WNT signaling is the principal underlying pathway via APC gene, while defective mismatch repair genes and epigenetic changes also are known to contribute.

**Case presentation:**

Here, we present an unusual case of rectal adenocarcinoma in a woman, with germline MSH6 and PMS1 mutations, and simultaneous somatic APC and TP53 mutations treated with surgery and adjuvant capecitabine.

**Conclusions:**

The case is unique suggesting a possible interaction between the two pathways and contributing to carcinogenesis in this patient. This also suggests need for a thorough germline and somatic mutation evaluation in select colorectal cancer patients to direct a tailored therapy.

## Background

Colorectal carcinoma (CRC) has a global incidence of 10% ranking third among all cancers overall and accounts for 9.4% of cancer deaths worldwide. It ranks third in males and is second only to breast cancer in females [1]. It is a heterogenous disease evolving from following main pathways: chromosomal instability (CIN) and microsatellite instability (MSI) which may be via CpG island methylator phenotype (CIMP) or defective mismatch repair (dMMR) genes. Majority of CRC are sporadic, while those arising due to dMMR have familial predisposition to cancer as in hereditary non-polyposis colorectal cancer (HNPCC) or Lynch syndrome (LS). Irrespective of the pathway and precursor, multiple mutations and epigenetic events are essential for carcinogenesis.

WNT signaling is the principal pathway underlying colorectal carcinoma in which APC gene inactivation (5q21) is the initiating event which may be germline (inherited as in FAP) or somatic (acquired). A total of 70–85% of CRC show widespread CIN [2] starting from APC gene inactivation leading to aberrant crypt formation (ACF) and progressing to adenoma and cancer via collective acquirement of activating mutations in oncogenes (KRAS, PIK3CA) and inactivation of tumor suppressor genes (SMAD4 and TP53) [3]. Around 15% CRC are euploid with numerous insertion/deletion loops (InDeLs) or mutations causing alteration in length of microsatellite allele (MSI) as a result of defective DNA mismatch repair. This may either be sporadic due to CpG island hypermethylation of MLH1 promoter region (CIMP) or inherited as in Lynch syndrome (2–3%) [4] due to germline mutations in mismatch repair genes (MLH1, MSH2, MSH6, PMS2, MLH3, MSH3, PMS1, EPCAM).

Here, we present an interesting case where both germline and sporadic pathways coexist with possible interplay which has not been reported prior. It further emphasizes the need for genetic evaluation and research to understand colorectal carcinogenesis.

## Case presentation

A woman in her 60 s presented with bleeding per rectum for 6 months. It was not associated with pain in abdomen or alteration in bowel habits. There was no family history of cancer in first- or second-degree relatives. On examination, abdomen was soft non-tender. On per rectal examination, an ulcero-proliferative growth was present 2.5 cm from anal verge along right anterolateral aspect. Upper limit of the growth could not be reached. There were no inguinal or supraclavicular lymph nodes.

**Fig. 1:**
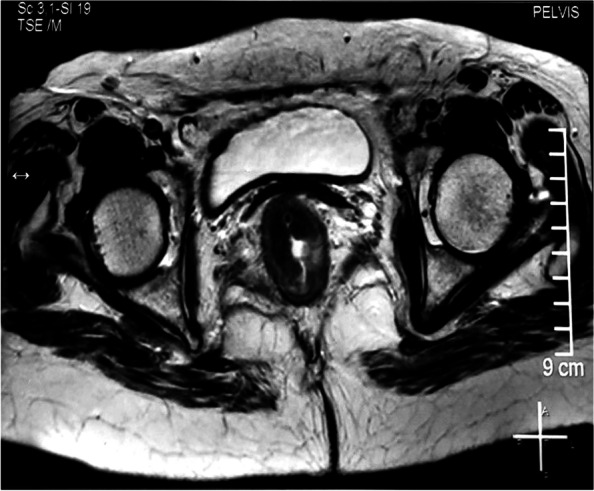
Axial view of MRI pelvis. Eccentric wall thickening (16 mm) involving distal rectum at right anterolateral aspect with heterogenous signal intensity, restricted diffusion and transmural extent, mesorectal fascia appears free, no significant locoregional lymph nodes

**Fig. 2:**
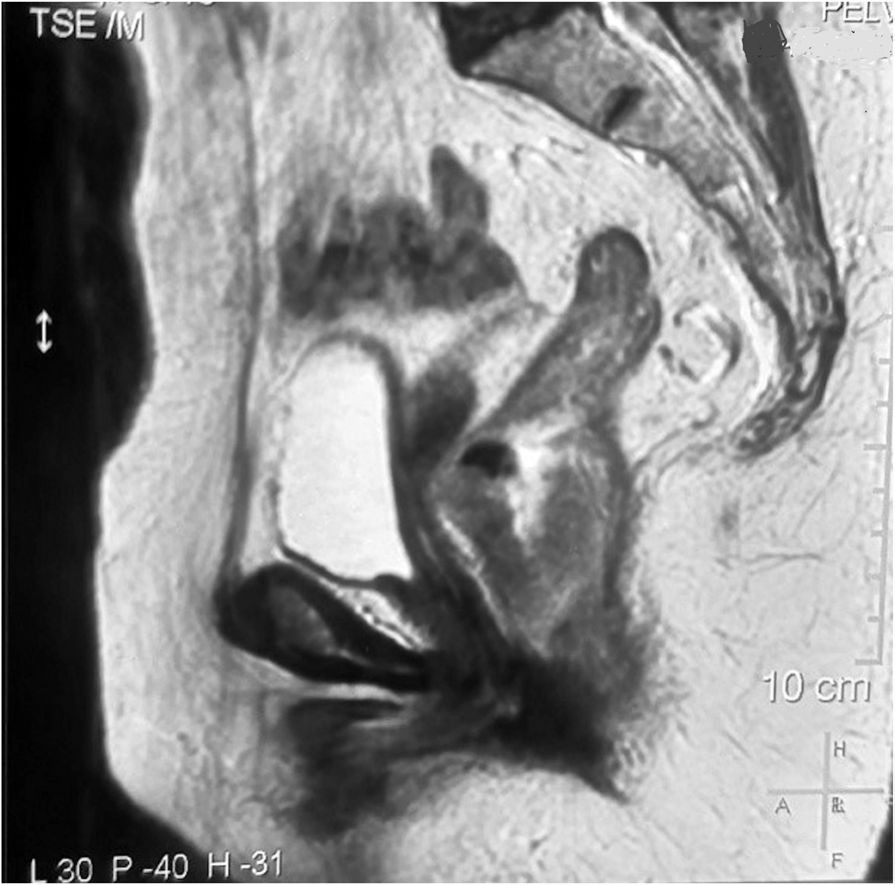
Sagittal view of MRI pelvis. Lesion extends from 26 mm proximal to anal verge and measures 52 mm in cranio-caudal dimension, planes with urinary bladder and uterus well maintained

To establish diagnosis, punch biopsy was done which revealed well-differentiated adenocarcinoma. Staging was done using CT scan of thorax abdomen and pelvis with MRI pelvis. Eccentric transmural thickening (16 mm) involving distal rectum and anal canal for 52 mm was noted 26 mm proximal to anal verge with maintained fat planes, clear circumferential radial margin (CRM), and no significant lymph nodes (cT2N0M0) (Figs. 1 and 2). Colonoscopy did not show any other synchronous lesion.

Patient underwent abdominoperineal resection (APR). Histopathology showed 4-cm annular ulcero-proliferative growth 2.5 cm from anal verge, well-differentiated adenocarcinoma reaching up to muscularis, all margins free, and 0/4 lymph nodes (pT2N0M0 stage I as per AJCC 8th edition).

The specimen was screened for mutations in 300 cancer genes with next-generation sequencing (NGS) using Ion AmpliSeq Cancer Hotspot Panel v2. It identified pathogenic mutations in TP53 (exon 7 p.Arg248Trp) and APC (exon 16 p.Glu1309fs) genes which point towards progression on adenoma-carcinoma sequence. Furthermore, tumor testing for microsatellite instability assay using BAT25, BAT26, NR21, NR22, and NR24 showed it to be microsatellite stable (MSS). Also, the patient was assessed for germline mutations via NGS platform (SMSEQTM) for 98 genes associated with hereditary cancers (Oncopro Hereditary Cancer Risk). This result revealed mutations in genes MSH6 (c.2039C > T; p.Ala680Val) and PMS1 (c.321A > C; p.Leu107Phe)[novel mutation not reported before]. The significance of these variants however has not yet been established. But both being DNA mismatch repair genes, their protein expression may be defective, and hence, the patient might have an increased predisposition to cancer (Lynch syndrome). Family members including siblings and children were counselled for increased risk of malignancy and advised hereditary cancer risk gene panel.

Owing to the presence of somatic APC and TP53, and germline MSH6 and PMS1 mutations in the patient, six cycles of single-agent capecitabine were given as adjuvant therapy. The patient was evaluated 2 months after completion of chemotherapy with PET scan and CEA which were normal. Patient was kept on follow-up at 3-month interval with CEA. Patient has been disease free for 36 months until date since the end of primary treatment.

## Discussion

Carcinogenesis involves a series of events through acquisition of multiple genetic alterations which makes the milieu susceptible for further mutations and the precancerous cells genomically instable accelerating the process [5]. Majority of the CRC have CIN leading to changes in chromosome number or structure (aneuploidy) [6] due to gain, loss, or translocation. This pathway involves hyperactivation of WNT signaling pathway mostly (80%) via inactivating mutation of APC gene, a negative regulator (80%), or alterations in other components like β-catenin (5–10%) [7]. It may proceed with activation of KRAS oncogene, loss of 18q (DCC, SMAD2, SMAD4), and loss of TP53 expression. However, only few cases have all chromosomal anomalies [8].

In our case, we identified somatic mutations in APC (exon 16,5q22.2 NM_000038.6: c.3927_3931delAAAGA [1] p.Glu1309fs) and TP53 (exon 7,17p13.1 NM_000546.6:c.742C > T p.Arg248Trp). The former allelic variant reported with a frequency of 60% resulted from a frameshift insertion and deletion which might lead to truncated or absent APC protein. The latter allelic variant with a frequency of 51% has a missense mutation. Both the mutations have been established as pathogenic as per ClinVar database, and data from numerous affected families in literature testify the same. Their coexistence support carcinogenesis along sporadic pathway.

Interestingly, screening for hereditary cancer genes revealed the presence of missense mutations in DNA mismatch repair genes — MSH6 (c.2039C > T) and PMS1 (c.321A > C), whose significance has not yet been established. MMR system functions to identify and correct errors during DNA replication-single base pair mismatch and insertion-deletion loops. HNPCC is a genetically acquired autosomal dominant disease resulting from germline defect in MMR genes which increases susceptibility to CRC as well as extra-colonic malignancies. It has incomplete penetrance and a variety of expressions depending upon the gene mutated. Mutations in MSH2 and MLH1 account for 80%, with MSH6 gene mutated in 7–20% cases, PMS2 in < 5% cases, and other MMR genes are involved rarely [9].

MSH6 mutations are associated generally with endometrial cancer and increase the likelihood of colorectal cancer by eightfold [10]. Pathogenic variants of MSH6 produce colorectal cancer at an older age in contrast to probands with MSH2 or MLH1 mutations. MSH6 forms a heterodimer with MSH2 (hMutS alpha), recognizes single pair mismatches and small InDeLs, and corrects them [11]. Here, a germline missense variant c.2039C > T was identified in coding exon 4 of MSH6 gene leading to alanine substitution by valine at codon 680 and alteration in a conserved residue in the protein. This variant has been reported in dbSNP database with identification number rs1558664035 and in Genome Aggregation Database (gnomAD) as a rare variant with frequency < 0.01%. It has not been shown to have clinical significance until date as per ClinVar database (VC000651717.4) in regard to hereditary cancer as per in silico prediction models. However, records of it have been submitted in ClinVar (accession: SCV0011748.2, SCV000947173.3, SCV001348668.2). Further studies are needed to establish its importance in disease causation.

PMS1, another MMR protein, forms complex with MLH1 (hMutL beta), hMutS complex, and other proteins to facilitate excision of incorrect nucleotides and their repair [12]. Its mutation has been found to be associated with LS in very few cases [13–15]. In this case, we report a novel heterozygous missense mutation in PMS1 gene, c.321A > C, p.Leu107Phe, in exon 4 on chromosome 2 which alters a conserved residue of protein. It has been found to be damaging by 2 out of 5 in silico missense prediction tools (FATHMM and Mutation Taster). It may point to an underlying susceptibility for LS. PMS1 is not classically implicated in LS and not routinely evaluated. Therefore, elaborate studies are required to establish its role in tumor development.

Microsatellite instability (MSI) testing is essentially important in phenotyping colorectal cancers and is indicated in all colorectal tumors. Instability results either due to defect in MMR gene or epigenetic silencing of promoter region mostly MLH1. Tumors with MSI have better prognosis compared to microsatellite stable (MSS) tumors. Although they do not respond to 5-fluorouracil treatment, they are amenable to immunotherapy. It can be assessed by two ways: PCR and IHC. In the former, nowadays, instability at five quasi-monomorphic microsatellite sites can be detected simultaneously: BAT-26, NR-21, BAT-25, MONO-27, and NR-24 [16]. In the present scenario, the patient was screened using the markers BAT-25, BAT-26, NR21, NR22, and NR24 and was found to be MSS. A study by Schiemann U. et al. [17] proposed a protracted marker panel (BAT40, D10S197, D13S153, D18S58, MYCL1) for reassessment of MSS tumors and predicting the presence of MSH6 mutations which might be actually MSI-L. All MSH6 mutations may not be detected either by PCR or IHC or both [18–20], and hence, NGS may be an essential tool in these cases.

## Learning points

- The current case had somatic APC and TP53 pathogenic mutations while also possessing germline MSH6 and PMS1 mutations suggesting progression of cancer along somatic pathway with underlying defective mismatch repair pathway as in LS.
- The late onset and slow progression could probably be due to low penetrance of dMMR genes which affects the phenotype.
- The case is unique in its evolution predicting coexistence of three different pathways of carcinogenesis and their possible interplay which has not been reported.
- An extended panel should be used for evaluating MSS tumors.
- In select cases, both germline and somatic mutations should be looked for as they will help in understanding of the disease process better.
- MSI testing using IHC or PCR may not be accurate, and NGS may be a better alternative

## Data Availability

All data analyzed during this study are included in this article. Further queries can be directed to the corresponding author.
